# Mycoplasma Contamination Revisited: Mesenchymal Stromal Cells Harboring *Mycoplasma hyorhinis* Potently Inhibit Lymphocyte Proliferation *In Vitro*

**DOI:** 10.1371/journal.pone.0016005

**Published:** 2011-01-11

**Authors:** Severin Zinöcker, Meng-Yu Wang, Peter Gaustad, Gunnar Kvalheim, Bent Rolstad, John T. Vaage

**Affiliations:** 1 Department of Immunology, Oslo University Hospital, Rikshospitalet and University of Oslo, Oslo, Norway; 2 Department of Anatomy, Institute of Basic Medical Sciences, University of Oslo, Oslo, Norway; 3 Institute of Tumor Biology, Oslo University Hospital, The Norwegian Radium Hospital, Oslo, Norway; 4 Institute of Microbiology, Oslo University Hospital, Rikshospitalet and University of Oslo, Oslo, Norway; 5 Institute of Cellular Therapy, Oslo University Hospital, The Norwegian Radium Hospital, Oslo, Norway; University of Muenster, Germany

## Abstract

**Background:**

Mesenchymal stromal cells (MSC) have important immunomodulatory effects that can be exploited in the clinical setting, *e.g.* in patients suffering from graft-*versus*-host disease after allogeneic stem cell transplantation. In an experimental animal model, cultures of rat T lymphocytes were stimulated *in vitro* either with the mitogen Concanavalin A or with irradiated allogeneic cells in mixed lymphocyte reactions, the latter to simulate allo-immunogenic activation of transplanted T cells *in vivo*. This study investigated the inhibitory effects of rat bone marrow-derived MSC subsequently found to be infected with a common mycoplasma species (*Mycoplasma hyorhinis*) on T cell activation *in vitro* and experimental graft-*versus*-host disease *in vivo*.

**Principal Findings:**

We found that *M. hyorhinis* infection increased the anti-proliferative effect of MSC dramatically, as measured by both radiometric and fluorimetric methods. Inhibition could not be explained solely by the well-known ability of mycoplasmas to degrade tritiated thymidine, but likely was the result of rapid dissemination of *M. hyorhinis* in the lymphocyte culture.

**Conclusions:**

This study demonstrates the potent inhibitory effect exerted by *M. hyorhinis* in standard lymphocyte proliferation assays *in vitro*. MSC are efficient vectors of mycoplasma infection, emphasizing the importance of monitoring cell cultures for contamination.

## Introduction

Mesenchymal stromal cells (MSC) comprise a heterogeneous population of progenitor cells that can differentiate along mesodermal lineages [1]. As characteristic markers are lacking, MSC are currently defined by a set of minimal criteria based on their morphology, phenotype and multipotency [2]. Multipotent stromal progenitor cells were originally isolated from the bone marrow (BM) [3], but are readily available from many adult and fetal tissues [4]–[7]. In recent years, a number of studies have shown that MSC have important immunomodulatory potential [8]–[12]. MSC can suppress the activation and proliferation of T and B cells, inhibit proliferation and cytotoxicity of NK cells, block the activation and maturation of dendritic cells, and induce expansion of regulatory T cells [13]. Although a variety of soluble mediator molecules have been implicated [14], the molecular mechanisms by which MSC exert their immunomodulatory effects are presently not well understood.

Graft-*versus*-host disease (GvHD) is caused by activation of donor T cells due to disparities of major histocompatibility complex (MHC) and minor histocompatibility antigens with the recipient. MSC have emerged as a promising treatment modality for GvHD after allogeneic stem cell transplantation [15], [16]. A clinical phase II study showed that a majority of patients suffering from acute, steroid-refractory GvHD responded to treatment with one or several MSC transfusions [17]. This effect was independent of the MHC constitution of the MSC donor. Conversely, results from experimental animal models have been conflicting. While some attempts to treat GvHD with MSC have been successful in murine models of allogeneic BM transplantation (BMT) [18], [19], other studies have failed to show a protective effect [20]–[22]. The influence of source of MSC, experimental protocols and treatment strategies on the efficiency of MSC remain unclear.

Mycoplasmas are parasitic bacteria that lack a cell wall and colonize animals and plants. In humans and other mammals, they have been associated with a variety of maladies in the respiratory tract and the genito-urinary tract. Interactions of mycoplasma with the host immune system are currently not fully understood. Efforts to prevent mycoplasma infection have proven difficult, partly due to the lack of successful vaccination strategies [23]. Several strains of mycoplasma frequently occur as latent contaminants of human and animal cell lines in research laboratories [24]. *Mycoplasma hyorhinis* is a pathogen of the porcine respiratory system and one of the most common cell culture contaminants [24]. Infections can remain undetected unless methods such as polymerase chain reaction (PCR) using mycoplasma-specific primer sequences are employed. Recently, *M. hyorhinis* has been implicated in the transformation of human prostate cells [25] and thus may pose a significant health risk related to carcinogenesis.

The aim of this work was to evaluate the immunosuppressive effects of rat MSC on mixed lymphocyte reactions (MLR) *in vitro* and GvHD *in vivo* using an experimental animal model of MHC-mismatched BMT. In the course of this work, we noted that our MSC lines were unusually potent inhibitors of MLR *in vitro* and discovered that this was caused by accidental contamination of the primary MSC cultures with *M. hyorhinis*.

## Results

### Mycoplasma*-*infected MSC strongly inhibit MLR and mitogen-induced T cell proliferation *in vitro*

**Figure 1:**
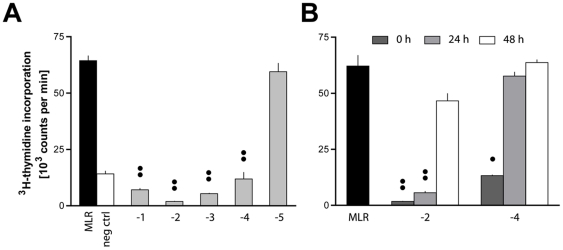
Mycoplasma-infected MSC effectively inhibit MLR. (**A**) MSC generated from the BM of PVG rats, subsequently found to be infected with *M. hyorhinis,* were added in tenfold dilutions at the start of co-cultures of 2×10^5^ PVG.7B LNC and 2×10^5^ irradiated BN LNC (gray bars). No MSC were added to the positive control (MLR, black bar). The basal proliferation of PVG.7B LNC in the absence of stimulus is also shown as negative control (neg ctrl, white bar). Values on the *x-*axis denote the common logarithm (log) of MSC:LNC ratios, *i.e.* 1∶10 (−1) through 1∶100 000 (−5). [^3^H]TTP incorporation was abrogated depending on the number of mycoplasma-infected MSC added. (**B**) MSC were added either at the start of MLR (dark gray bars), after 24 h (light gray) or 48 h (white), respectively, at 1∶100 (−2) or 1∶10 000 (−4) MSC:LNC ratios, and co-cultured for a total of 4 d. No MSC were added to the positive control (MLR). Mycoplasma-infected MSC fully inhibited the assay with at least 3 d of co-incubation at the 1∶100 MSC:LNC ratio. Representative data from at least three independent experiments are shown as the mean plus the standard error of the mean of triplicates. Statistical difference to the respective positive controls, • *P*<.05, •• *P*<.01.

A MSC line generated from the BM of PVG strain rats (expressing the *RT1^c^* MHC haplotype) exhibited a potent inhibitory effect on lymphocyte proliferation *in vitro* (Figure 1). Tenfold dilutions of MSC were added to a MLR of PVG.7B (*RT1^ c^*;used interchangeably with PVG) lymph node cells (LNC) and allogeneic irradiated BN (*RT1^n^*) stimulator cells. Incorporation of tritiated thymidine ([^3^H]TTP) was inhibited at ratios up to 1 MSC per 10 000 responder cells (10^−4^) (Figure 1A). MSC were equally effective inhibitors of proliferation induced by Concanavalin A (Con A; data not shown). Irradiation of MSC (20 Gy) did not change their inhibitory capacity (data not shown). MSC showed a marked inhibitory effect when introduced at delayed time points during MLR or mitogen-induced stimulation, but required at least 3 d of co-incubation with the responder cells (Figure 1B and data not shown).

**Figure 2:**
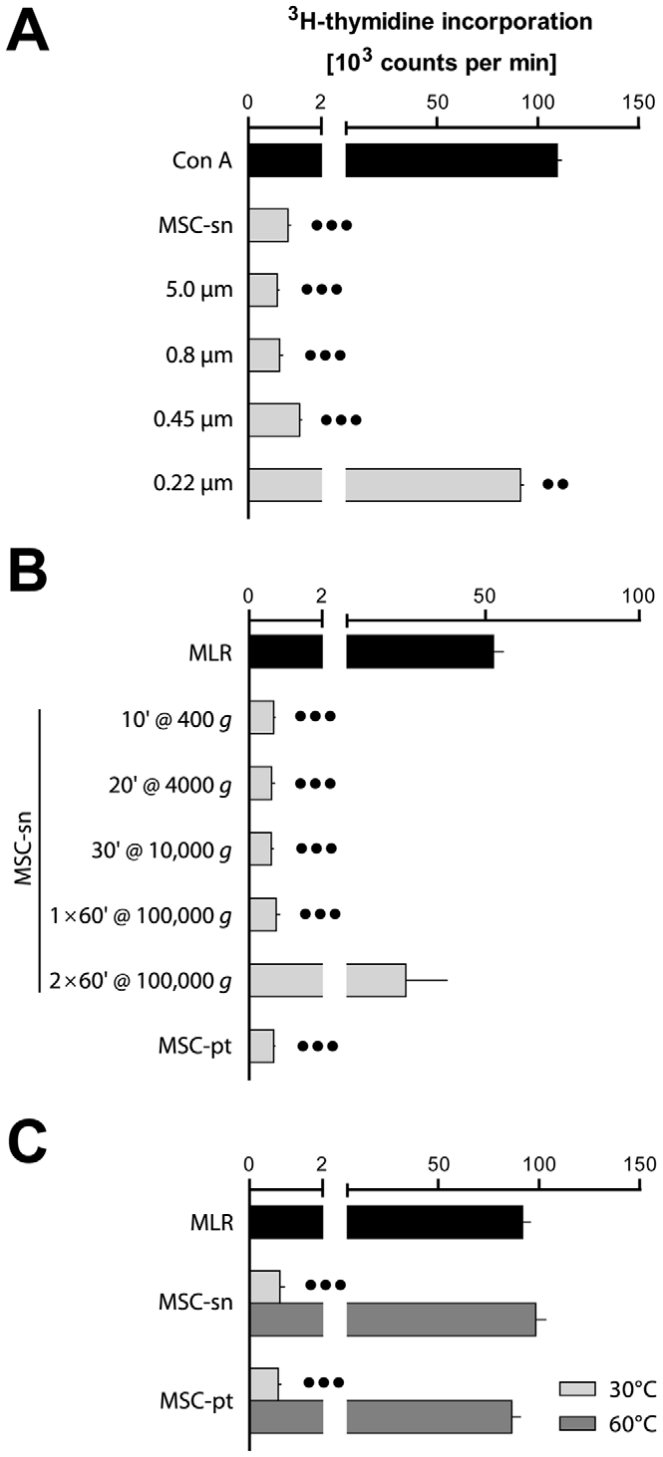
MLR is inhibited by MSC culture supernatant. Inhibition of lymphocyte proliferation was reversed either by (**A**) filtration at 0.22 µm, (**B**) serial centrifugation at 100 000 *g*, or (**C**) heat-treatment at 60°C. (**A**) MSC-conditioned supernatant (MSC-sn; gray bars) was added at 1∶10 (v/v) dilution (22 µL added to 200 µL per well) at the start of mitogenic stimulation of 2×10^5^ PVG.7B LNC with Con A (positive control with no MSC-sn added, black bar). MSC-sn was added either unfiltered or filtered with the indicated cut-off sizes. (**B**) MSC-sn was sedimented with the indicated centrifugal forces and durations (min) and added at 1∶10 (v/v) dilution at the start of allogeneic MLR (cf. Figure 1). The pellet fraction (MSC-pt) was obtained after centrifugation twice for 60 min at 100 000 *g*, resuspended and added at 1∶10 (v/v) dilution to the MLR. (**C**) Unprocessed MSC-sn or the resuspended pellet fraction described in panel B were treated at 30°C (light gray bars) and 60°C (dark gray) for 30 min, respectively, before adding to MLR (dilution 1∶10). Representative data of at least two independent experiments are shown as mean values plus the standard error of the mean of quadruplicates. Statistical difference to the respective positive controls, •• *P*<.01, ••• *P*<.001.

MSC exerted a potent inhibitory effect in transwell co-cultures with LNC using 0.4 µm pore size membranes (data not shown). Addition of cell-free MSC culture medium also resulted in strong inhibition of MLR, but this effect was reversed by filtering through 0.22 µm membranes or repeated centrifugation at 100 000 *g* (Figure 2A,B). Inhibition was furthermore mediated by the pellet fraction sedimented at 100 000 *g*, but reversed by heat-inactivation for 30 min at 60°C (Figure 2B,C). These inhibitory effects were striking compared with several previous reports of MSC from different species [8], [9], [11], [12], [20], [21], and it was therefore with some disappointment that we discovered that the cells were infected with *M. hyorhinis*, a common cell culture contaminant (cf. Materials and Methods).

### A single mycoplasma-infected MSC can inhibit allogeneic MLR

**Figure 3:**
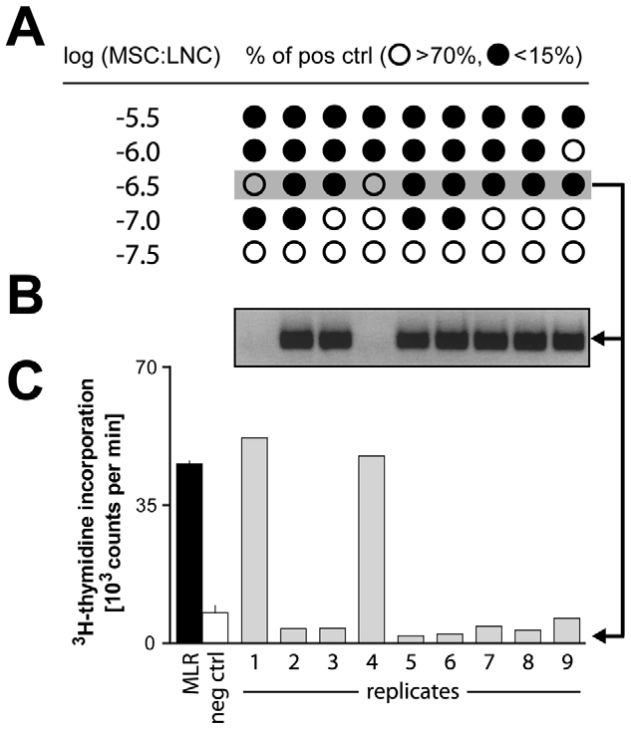
A single mycoplasma-infected MSC can completely block the MLR. (**A**) Mycoplasma-infected MSC were added at limiting dilution conditions at the start of MLR cultures with 2×10^5^ responder cells. Proliferation was assessed in 9 replicate wells for each dilution. Wells with more than 70% or less than 15% of the [^3^H]TTP incorporation observed in the positive control (MLR without MSC) are shown as open (**○**) and filled circles (•), respectively. The log of MSC:LNC ratios are denoted. (**B**) Mycoplasma was detected in the supernatant of seven out of nine wells at the −6.5 dilution shown in panel A, and in the same wells (**C**) complete inhibition of MLR was observed. Positive (MLR) and negative (neg ctrl) controls are also shown (mean of triplicates). Results are representative of three independent experiments.

A second MSC line derived from another MHC-congenic strain on the PVG background (PVG.1U expressing the *RT1^u^* MHC haplotype), which was also subsequently found to be infected with *M. hyorhinis*, displayed an even stronger inhibition of lymphocyte proliferation (Figure 3). [^3^H]TTP incorporation was fully abrogated (less than 15% of the positive control) by addition of only 2 MSC per 200 000 LNC responder cells (10^−5^) at the start of the MLR culture. At even lower MSC:LNC ratios (10^−6^), we observed an “all-or-none” phenomenon, with either full inhibition or normal proliferation in individual wells. To evaluate whether inhibition was mediated by single infected cells, we added serial threefold dilutions in replicate wells as depicted schematically in Figure 3A. Complete block of proliferation was observed in mycoplasma-contaminated wells as detected by PCR, while normal responses were recorded in mycoplasma-negative wells (Figure 3B,C). Analogous results were obtained for MSC/LNC co-cultures stimulated with Con A (data not shown). These data suggest that *M. hyorhinis* has an extremely potent inhibitory effect on lymphocyte proliferation *in vitro*.

### The suppressive effect of mycoplasma infection is removed by anti-mycoplasma treatment and restored by re-infection

**Figure 4:**
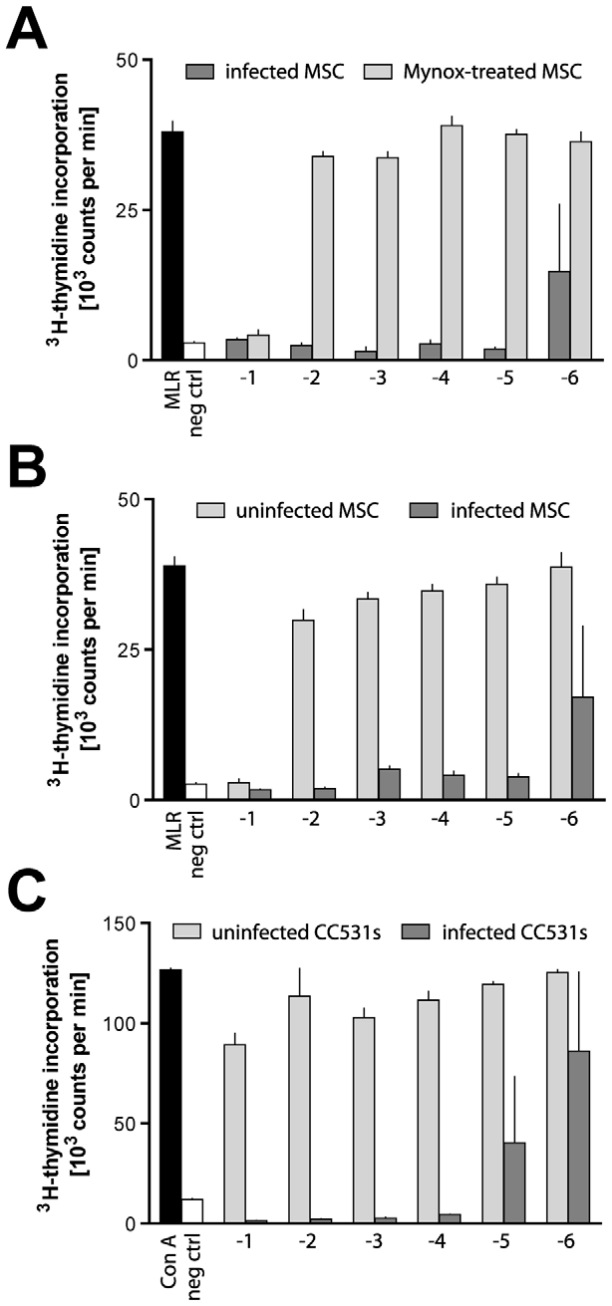
Mycoplasma-treated or previously uninfected MSC show a markedly reduced ability to inhibit lymphocyte proliferation. (**A**) Infected PVG.1U MSC were treated with Mynox reagent to eradicate the mycoplasma infection. Tenfold dilutions of untreated (dark gray bars) and treated MSC (light gray) were tested in allogeneic MLR. Mycoplasma-treated MSC, which tested negative for mycoplasma by PCR, inhibited MLR at 1∶10 (−1) but not at higher dilutions. Mycoplasma-infected MSC, on the other hand, effectively inhibited up to a cell ratio of 1∶10^6^ (−6). Previously uninfected PVG.7B MSC (**B**) or the rat colon carcinoma cell line CC531s (**C**) were intentionally infected with *M. hyorhinis* by transfer of cell culture supernatant from the infected PVG.1U MSC line shown in panel A. Infection was verified by PCR after passage. MSC were added at the start of MLR, and CC531s cells were irradiated to prevent spontaneous proliferation and added at the start of lymphocyte culture with Con A. Proliferation was effectively inhibited by addition of *M. hyorhinis*-infected cells, but not by uninfected CC531s cells. Values on the *x-*axis denote the log of MSC:LNC and CC531s:LNC ratios, respectively. When infected cells were added at the highest dilutions, individual wells showed either full inhibition or a normal proliferative response (cf. Figure 3); *e.g.* when infected CC531s cells were added to the Con A culture, proliferation was detected in one of three (log dilution −5) and two of three (−6) replicates. Representative data from at least three independent experiments are shown as the mean plus the standard error of the mean of (**A, B**) quadruplicates or (**C**) triplicates.

The PVG.1U MSC line was treated with Mynox (cf. Materials and Methods) to resolve the mycoplasma infection. As a result of the treatment, the cells were consistently free from mycoplasma as confirmed by PCR, and most of the observed inhibitory capacity was concomitantly lost (Figure 4A). MLR stimulation was prevented only at a relatively high MSC:LNC cell ratio of 1∶10, in line with several previous MSC studies [9], [20], [21]. Similar results were obtained with Con A-induced proliferation (unpublished observations). Intentional contamination of a previously uninfected MSC line with mycoplasma-containing supernatant increased its suppressive potential dramatically (Figure 4B), showing the same phenomenon of either full inhibition or a normal proliferative response in individual wells at limiting dilutions. Similar inhibitory potential was observed after deliberate infection of a rat colon carcinoma (CC531s) cell line (Figure 4C). These data were firm evidence that the strong inhibitory effect was mediated by the contaminant and was not dependent on a specific cellular vehicle.

### *M. hyorhinis* effectively inhibits T cell proliferation as evaluated by Carboxyfluorescein diacetate succinimidyl ester (CFSE) dilution assay

**Figure 5:**
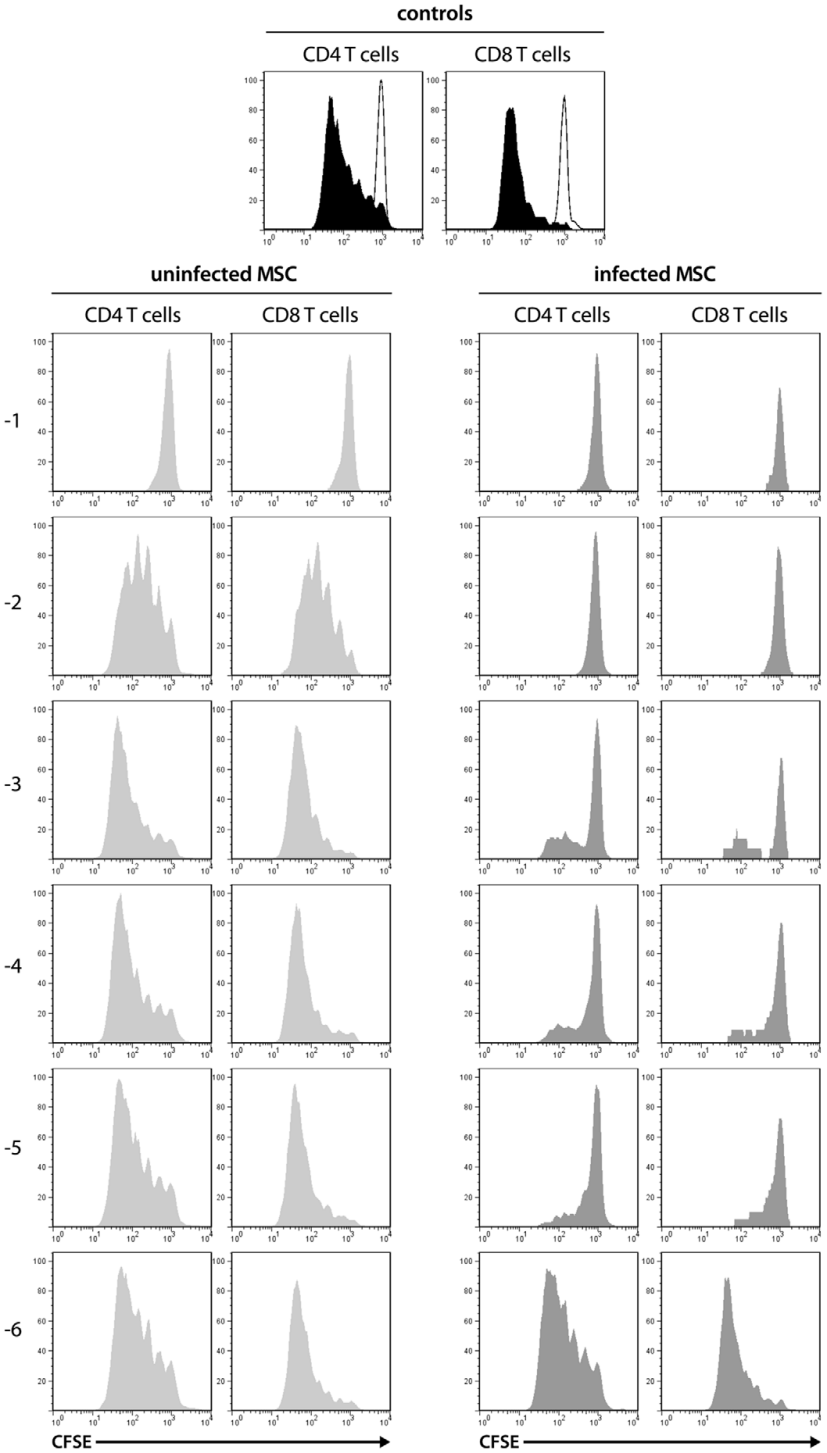
Mycoplasma-infected MSC inhibit T lymphocyte proliferation in vitro as measured by CFSE dilution. Previously uninfected (light gray histograms) and intentionally infected PVG.7B MSC (dark gray; cf. Figure 4B) were added to CFSE-labeled, Con A-stimulated PVG.7B LNC (values to the left denote the log of MSC:LNC ratios). LNC alone were cultured either with (black histogram) or without (white) Con A as positive and negative controls, respectively. CFSE dilution indicating cell divisions was measured by flow cytometry after 3 d of incubation. Histogram plots (percent of maximum count) are representative of triplicates and show the fluorescence intensity of lymphocyte populations gated on CD3^+^CD4^+^CD8^−^ (CD4 T cells) and CD3^+^CD4^−^CD8^+^ (CD8 T cells). The potent inhibition of lymphocyte proliferation by infected MSC measured by CFSE dilution cannot be explained by degradation of [^3^H]TTP. For the highest dilution (−6) of mycoplasma-infected MSC, individual cultures displayed either full inhibition or full proliferation (cf. Figure 3); one replicate in which full lymphocyte proliferation was detected is shown. Data are representative of three independent experiments.

Mycoplasmas are known to interfere with the read-out of [^3^H]TTP incorporation assays commonly used to measure DNA synthesis during lymphocyte proliferation, due to their endogenous pyrimidine-nucleosidase activity [26]. We therefore opted to use the CFSE dilution assay as an alternative method to measure cell division and cell proliferation [27]. Infected and mycoplasma-treated MSC were added to Con A cultures of CFSE-labeled LNC (Figure 5). The presence of mycoplasma-infected MSC led to increased cell division arrest of both CD4^+^ and CD8^+^ T cells compared to mycoplasma-free MSC. The levels of inhibition of cell division correlated with [^3^H]TTP incorporation of LNC performed in parallel, and similar results were obtained for allogeneic MLR (data not shown).

We also determined the relative numbers of natural T regulatory (T_reg_) cells in our assays. The frequency of CD4^+^CD25*^hi^*FoxP3^+^ T_reg_ cells markedly decreased as a result of addition of MSC to Con A cultures (Figure S1A), indicating that inhibition was not mediated by T_reg_ cells. In line with this observation, cell death was increased in mycoplasma-contaminated LNC cultures (Figure S1B). Taken together, these data show that *M. hyorhinis* has a high capacity to arrest lymphocyte proliferation *in vitro*. The extent to which *M. hyorhinis* interfered with the [^3^H]TTP incorporation assay by substrate degradation is not known, however, the results from CFSE dilution assays suggest that this effect is of less importance.

### Mycoplasma is rapidly disseminated in lymphocyte cultures

**Figure 6:**
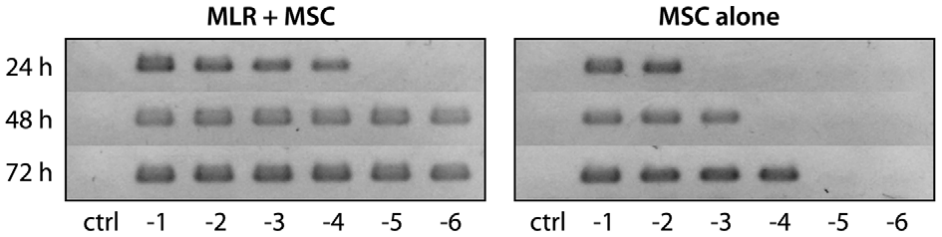
Mycoplasma infection spreads rapidly in MLR cultures. Mycoplasma-positive MSC were cultured either alone (right panel) or together with PVG responder cells and irradiated BN cells (left panel) for 3 d. Supernatants were collected at the indicated time points and tested by PCR. The log dilutions of MSC are denoted; ctrl, no MSC added. *Mycoplasma* was detectable at considerably lower MSC concentrations when co-cultured with lymphocytes.

Because cultivation of *M. hyorhinis* in cell-free anaerobic agar medium for microbiological assays is rarely feasible [24], we performed semi-quantitative measurements of bacterial load by PCR analysis of sequentially collected culture supernatants (Figure 6). Initially, mycoplasma was detected only at high MSC:LNC ratios, but bacterial titers increased more rapidly in co-culture with LNC as compared to cultures of MSC alone, and mycoplasma infection manifested even in the highest dilutions where only single infected MSC were added to the MLR. Similar results were obtained for PCR-testing of supernatants from Con A-stimulated LNC co-cultures with MSC (data not shown).

### Mycoplasma-infected MSC retain their cell phenotype, differentiation potential and cytokine expression profile in mixed lymphocyte cultures

Mycoplasma-contaminated MSC adhered to plastic surface *in vitro* and had the cell morphology of spindle-shaped colony-forming unit fibroblasts (Figure S2A). The infection did not seem to inhibit *in vitro* growth or the differentiation potential of MSC (Figure S2B,C,E-H and unpublished observations). MSC expressed surface markers CD59, CD71, CD90, and CXCR4, but lacked CD31 and CD45 (Figure S2D). Class I MHC molecules (RT1-A) were also expressed while class II MHC molecules (RT1-B/D) were not detected by flow cytometric staining. Based on these parameters, the cells are in accordance with the current definition of MSC [2].

**Figure 7:**
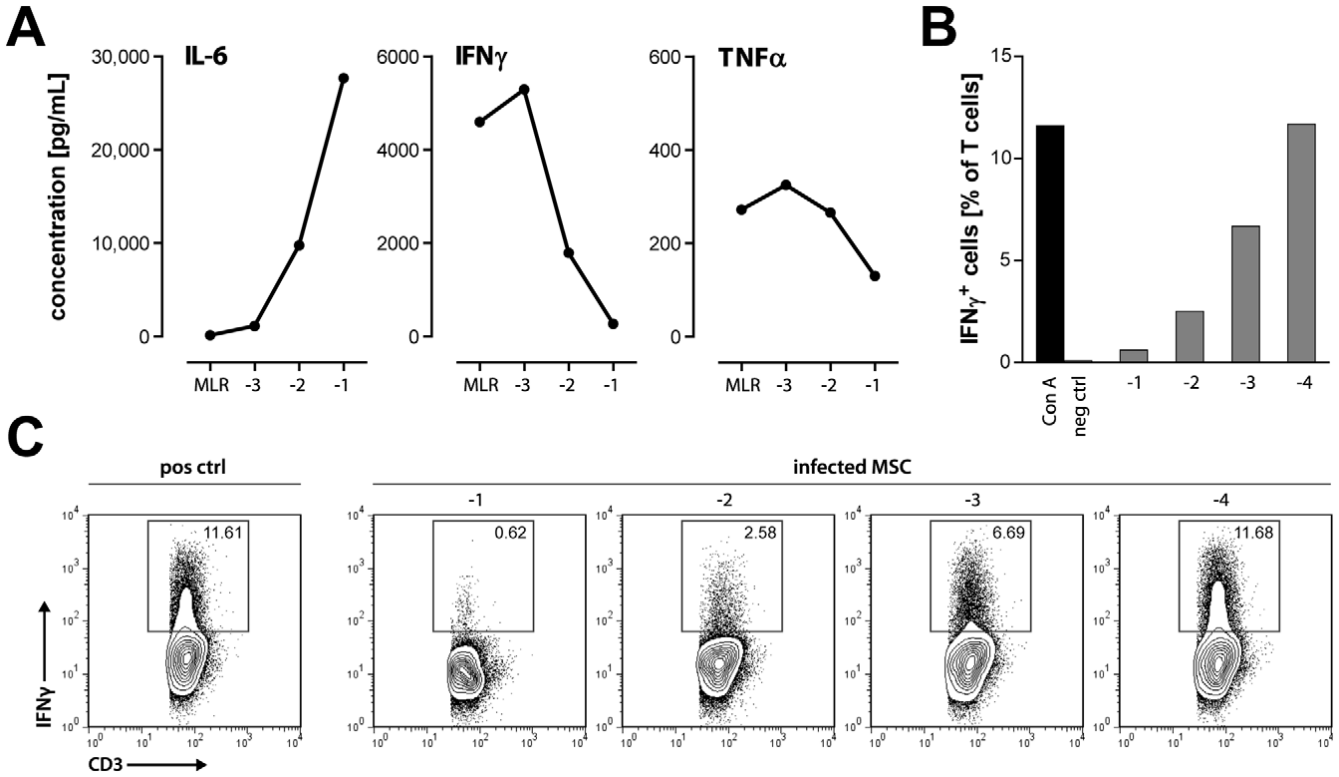
Mycoplasma-infected MSC alter the cytokine profile of MLR and inhibit IFNγ production by T cells. (**A**) Mycoplasma-positive MSC (PVG) were added at the start of MLR of PVG.7B and irradiated BN LNC (1×10^6^ cells each) at the indicated dilutions (log [MSC:LNC]). The concentration of various cytokines was determined in the supernatant at the end of 6 d co-culture. IL-6 was markedly increased, while interferon-γ (IFNγ) and tumor necrosis factor-α (TNFα) were reduced by addition of MSC compared to the control MLR. Representative data from three independent experiments are shown as the average of duplicates. (**B**) Mycoplasma-infected MSC (PVG.7B) were added at the start of Con A-stimulated culture of PVG.7B LNC at the indicated tenfold dilutions. The fraction of IFNγ-expressing CD3^+^ T cells was determined after 24 h by intracellular staining (triplicates were pooled) and flow cytometry, as shown in (**C**). Cells were gated on CD3^+^ lymphocytes, numbers represent the percentage of gated cells. Data are representative of three independent experiments.

Culture supernatants from mycoplasma-positive MSC alone contained considerable amounts of interleukin (IL)-6, but not other cytokines tested (Figure S3A), in agreement with previous observations of uninfected MSC [28]. Adding increasing numbers of mycoplasma-positive MSC to MLR resulted in no significant changes of the concentrations of IL-1β, IL-2 and IL-10. The levels of IL-1α, IL-6, and granulocyte macrophage colony-stimulating factor, however, were markedly increased, while IL-4, interferon-γ (IFNγ) and tumor necrosis factor-α were clearly diminished after 6 d of co-incubation (Figure 7A and data not shown). These results were in agreement with several previous observations on the effects of murine and human MSC on cytokine secretion by stimulated lymphocytes [29]–[31], but failed to reproduce an increase of IL-10 in co-cultures with human MSC [29], [32].

Next, we studied the capacity of *M. hyorhinis* to suppress the production of IFNγ as an important function of activated T cells. A significant fraction of T cells produce intracellular IFNγ after 24 h of Con A stimulation (Figure 7B,C). Addition of mycoplasma-infected MSC at ratios up to 1∶1000 reduced the relative frequency of IFNγ^+^ T cells. This effect can explain the concentration-dependent decrease of IFNγ observed in MLR/MSC co-cultures (Figure 7A).

Blocking the IL-6 signal by addition of IL-6 specific antibody did not counteract the suppressive effect of infected MSC. Also, addition of recombinant IFNγ, either alone or in combination with anti-IL-6, was without effect (Figure S3B). These data suggest that the changes in IL-6 or IFNγ were not important for the suppressive effect mediated by mycoplasma-infected MSC.

### Intravenous injections of mycoplasma-positive MSC fail to reduce GvHD severity in BM-transplanted rats

In a model of experimental allogeneic BMT, we used the same combination of PVG.7B donor rats and fully MHC-mismatched BN recipient rats as for MLR *in vitro* experiments. Lethally irradiated (9.0 Gy total body irradiation) BN rats were injected with 30×10^6^ T cell-depleted PVG.7B BM cells. Two weeks later, at a time point when the transplanted recipients had regained their initial body weight, donor lymphocyte infusions (DLI) of graded minimal doses of 1.5–2.5×10^6^ CD3^+^ T cells from PVG.7B lymph node donors reproducibly invoked lethal GvHD in 90% of the recipients. Two groups were treated with intravenous injections of high doses of MSC that later proved to be infected with *M. hyorhinis*.

Transplanted rats received either single or repeated injections of 1×10^6^ MSC 14 d after DLI before or after rats had developed symptoms of ongoing acute GvHD, but with no improvements of GvHD morbidity and mortality. All recipients were moribund between 14 and 26 d after DLI regardless of either repeated prophylactic (3× MSC) or acutely curative (1× MSC) cell therapy (for details refer to Figure S4).

## Discussion

We here report a potent inhibitory effect of *M. hyorhinis* infection of MSC on *in vitro* lymphocyte proliferation assays based on [^3^H]TTP incorporation and CFSE dilution. Addition of single infected MSC led to rapid dissemination of mycoplasma in mixed lymphocyte cultures. *Mycoplasma* spp. have been shown to reach titers in cell cultures sufficient to degrade pyrimidine substrates with more than 90% efficiency within 3–5 d [34]. In our study, high titers of mycoplasma were reached already after 2 d which may explain the observed potent inhibition of lymphocyte proliferation as a result of direct cytopathic/cytolytic effects. Studies testing for proliferative responses should therefore rigorously control their cell cultures for mycoplasma contamination.

Many mycoplasma strains, including *M. hyorhinis*, produce nucleoside phosphorylases which rapidly convert [^3^H]TTP to its thymine-derivative *in vitro* [26], [35]. Caution has therefore been advised in the interpretation of *in vitro* proliferation assays based on quantification of such compounds [26], [34]. Due to its potent enzymatic activity, it has been suggested that [^3^H]TTP degradation can be utilized as a sensitive detection method for mycoplasma infection [36], however, specific PCR and other readily available detection methods today have advantages of ease of use and relatively low cost. We did not directly assess to which extent our results reflect degradation of pyrimidine substrate, but this mechanism could not explain the observed effect as proliferation was similarly inhibited when measured by CFSE dilution.

These experiments also showed that proliferation of both CD4^+^ and CD8^+^ T cells was equally abrogated, and were in agreement with the finding that ovine Con A-activated lymphoblast cultures were inhibited by a different mycoplasma species (*M. ovipneumoniae)* in a recent study [37]. CD4^+^ T cells are critically involved in clearing persisting bacterial infection. We speculate that the ability to directly infect proliferating T lymphocytes inducing cell cycle arrest and cell death can be an important mechanism for mycoplasmas to evade and counteract an immune response. Other plausible mechanisms such as inhibition through mycoplasma membrane proteins have been suggested [38], [39].

The inhibitory properties of rat MSC were dramatically reduced by removal of *M. hyorhinis* by anti-mycoplasma treatment. Mycoplasma-negative MSC had a weaker, but significant inhibitory effect on T cell proliferation. Their suppressive potential is in agreement with several previous findings where MSC inhibition of responder cells were measured at ratios of 1∶1 to 1∶100 [8], [9], [11], [12], [20], [21], while some studies have reported exceptionally high immunosuppressive potential of MSC [10], [40], [41].

In contrast to their substantial suppressive effects on lymphocytes *in vitro*, mycoplasma-infected MSC did not have a beneficial effect upon preemptive or curative treatment of BM-transplanted rats suffering from acute GvHD. The applied dosage of 1–2×10^6^ MSC (approximately 4–10×10^6^ per kg body weight) is comparable to what is typically administered in patients [16], [17]. Increasing the dose above 2×10^6^ MSC per injection was associated with a high risk of pulmonary embolism and sudden death of the animals (unpublished observations). Injected MSC may be susceptible to immune responses in the host against mycoplasma infection, which could explain their lack of efficiency in alleviating GvHD. We cannot rule out, however, that the MSC lines applied in the present study are ineffective in suppressing alloreactive donor lymphocytes *in vivo*, as has been reported for other animal studies [20]–[22], [42]. The extent to which *M. hyorhinis* infection can affect the inhibitory properties of MSC *in vivo* is therefore not clear.

In our hands, mycoplasma infection did not deprive MSC of their characteristic differentiation potential, nor their capacity to proliferate or to secrete cytokines. Infection did not result in an upregulation of class I or class II MHC surface expression. Thus, *M. hyorhinis* infection of MSC may pass unnoticed. The host-pathogen relationship between *M. hyorhinis* and MSC remains elusive. The two important pro-inflammatory cytokines IFNγ and tumor necrosis factor-α were strongly reduced by the addition of infected MSC to mixed lymphocyte cultures [29]–[31]. It is conceivable that *M. hyorhinis* can alter the inherent immunosuppressive potential of MSC [43], e.g. by changing the expression of cytokines and other important secreted proteins [44], [45]. In order to dissect such mechanisms, it will be necessary to gain a better understanding of the immunomodulatory properties of BM stromal progenitor cells as well as the pathobiology of mycoplasma infection on MSC and immune cells. This research may have important implications for the design of vaccination strategies against mycoplasma species which continue to cause disease requiring extensive use of antibiotics in veterinary and human medicine.

## Materials and Methods

### Ethics statement

Our Department of Comparative Medicine at the Institute of Basic Medical Sciences led by our institutional veterinarian has established the rules for feeding, monitoring and handling of laboratory animals in compliance with regulations set by the Ministry of Agriculture of Norway. The institutional veterinarian has delegated authority from the Norwegian Animal Research Authority (NARA) and approved the protocols (license numbers: VIT02.02, VIT09.1512, 05.07), which were conducted in compliance with “The Norwegian Regulations on Animal Experimentation” and “The European Convention for the Protection of Vertebrate Animals used for Experimental and other Scientific Purposes”. Intravenous injections were performed under neuroleptanalgesia with fentanyl citrate and fluanisone (Hypnorm®; VetaPharma, UK). All animals used in this study were euthanized with CO_2_, and every effort was made to minimize their suffering.

### Animals

The PVG-*RT7^b^* (PVG.7B) strain expresses an allotype of rat CD45 (RT7.2) and was used interchangeably with the standard PVG strain (RT7.1) as both strains express the *RT1^c^* haplotype of the rat MHC. PVG.7B and PVG-*RT1^u^* (PVG.1U; *RT1^u^*) rats were bred at the Institute of Basic Medical Sciences, University of Oslo. PVG and BN (*RT1^n^*) rats were purchased from Harlan, The Netherlands. The animals were housed on location under a 12∶12 h light/dark cycle with access to food and filtered drinking water *ad libitum*, and were routinely screened for common pathogens following FELASA recommendations [46].

### MSC isolation, *ex vivo* culture and differentiation assays

MSC lines were obtained from 8–11 w-old PVG, PVG.7B and PVG.1U strain rats as previously described [47]. In short, BM cells were aspirated from femurs and tibias, filtered through nylon cell strainers (70 µm mesh; BD Biosciences, MA), and cultured in MSC medium comprising α-modified minimal essential medium supplemented with 20% fetal bovine serum and 100 U mL^−1^ penicillin, 100 µg mL^−1^ streptomycin, 250 ng mL^−1^ amphotericin B (all from Invitrogen, UK) and 2 mM L-glutamine (Millipore, MA) in 175 cm^2^ culture flasks (Nunc, Denmark) at 37°C in a humidified atmosphere of 5% CO_2_. Non-adherent cells were removed after approximately 24 h by replacement of the MSC medium. Adherent cells were allowed to expand to near confluence, detached using 500 µg mL^−1^ trypsin and 200 µg mL^−1^ EDTA•4Na (Invitrogen) and reseeded at a density of approximately 500 cells per cm^2^. Cells were used in experiments after the third passage.

The differentiation potential of MSC was tested using adipogenic and osteogenic assays. MSC were cultured for 3 w in MSC medium supplemented with adipogenic (StemCell Technologies, BC) or osteogenic (10 nM dexamethasone, 50 µg mL^−1^ ascorbic acid, and 5 mM β-glycerophosphate; StemCell Technologies) stimulatory supplements. Neutral lipids in fat vacuoles were visualized by staining with Oil Red O (Sigma Aldrich, CO) and mineralization by staining with 40 mM Alizarin Red (Sigma Aldrich).

### Mycoplasma detection, treatment and infection

Supernatants from confluent cultures were tested for mycoplasmas by nested PCR [48] using the following primer sequences: Mike-O-Plasma-F1 5′-ACACCATGGGAGCTGCTAAT-3′; Mike-O-Plasma-R1 5′-CTTCWTCGACTTYCAGACCCAAGGCAT-3′; Mike-O-Plasma-F2 5′-GTTCTTTGAAAACTGAAT-3′; Mike-O-Plasma-R2 5′-GCATCCACCAWAWACTCT-3′. Mycoplasma-positive PCR test results were confirmed using a detection kit (MycoAlert®; Lonza Rockland, ME) based on enzymatic ATP-conversion combined with luminescence measurement. MSC that tested positive for mycoplasma were cultured in a quarantine laboratory using MSC medium without antibiotics.

The mycoplasma strain was detected and identified as *M. hyorhinis* from cell cultures by means of 16S ribosomal DNA PCR and sequencing. DNA extracted from the cell cultures was amplified and sequenced (ABI Prism 3730 DNA analyzer; Applied Biosystems, CA) using two primers within the 16S rRNA gene. The obtained sequences were compared to sequences in the NCBI database using BLAST version 2.210.

In some experiments, non-infected MSC lines (PVG.1U or PVG.7B) and a rat colon carcinoma cell line (CC531s, a kind gift from Peter Kuppen, Leiden, The Netherlands) were deliberately infected with *M. hyorhinis* by transferring membrane-filtered (0.45 µm Filtropur S; Sarstedt, Germany) culture supernatant from nearly confluent cultures of mycoplasma-positive MSC and incubated for 1–2 d before replacement of fresh culture medium. Infected cells were confirmed as mycoplasma-positive by PCR. CC531s cells were maintained in culture medium comprising Dulbecco's modified Eagle's medium (Invitrogen) supplemented with 20% fetal bovine serum (Invitrogen) without antibiotics in a quarantine laboratory. In another experiment, the original infected MSC line was cleared of mycoplasma using Mynox® reagent (Minerva Biolabs, Germany) according to the manufacturer's protocol, and was confirmed negative by PCR. The treated cell line continued to grow normally and no recurrence of mycoplasma was observed in several passages after the treatment.

### Mixed lymphocyte cultures and radionucleotide incorporation assays

Mononuclear cells from mesenteric and cervical lymph nodes of 8–14 w-old PVG, PVG.7B and BN rats were obtained by filtering through nylon cell strainers (BD Biosciences) and density gradient centrifugation (Lymphoprep™; Medinor, Norway) following the manufacturer's protocol. Stimulator cells were irradiated with a dose of 20.0 Gy using a ^137^Cs source (Gammacell® 3000; MDS Nordion, Canada). MLR were performed in RPMI medium 1640 supplemented with 10% heat-inactivated fetal bovine serum, 100 U mL^−1^ penicillin, 100 µg mL^−1^ streptomycin, 2 mM L-glutamine and 25 µM 2-mercaptoethanol (MLR medium; all from Invitrogen) with responder (PVG or PVG.7B) and stimulator cells (BN; each 2×10^5^ unless otherwise specified) in 200 µL per well using round-bottom, 96-well cell culture clusters (Corning, NY). In the same format, Con A (Sigma Aldrich, MO) was used at a final concentration of 5 µg mL^−1^ for mitogenic stimulation of responder cells. Polyclonal rabbit anti-rat IL-6 antibody (PeproTech, UK) at 2 µg mL^−1^ or recombinant rat IFNγ at 500 U mL^−1^ was added where specified.

CC531s and MSC were harvested using trypsin and EDTA (Invitrogen), washed twice, diluted in MLR medium, and in some experiments irradiated (20 Gy) to inhibit mitosis, before adding to MLR cultures at the indicated cell ratios. Medium supernatants were removed from MSC cultures prior to cell harvest and sedimented at 400 *g* for 10 min before adding to MLR cultures at the indicated dilutions. They were further processed using syringe filter units (Millipore, Ireland) of different mesh size (indicated) or serial sedimentation applying increasing centrifugal force and durations (indicated). The pellet fraction of 100 000* g* sedimentation (Optima™ LE-80K Ultracentrifuge; Beckman Coulter Inc., CA) was resuspended in a volume of MLR medium equal to the original amount of supernatant.

DNA synthesis was assessed after 3 d of mitogen stimulation or 4 d of mixed lymphocyte culture by pulsing with 1 µCi of [^3^H]TTP (diluted in 20 µL MLR medium; Hartmann Analytic, Germany) 18–20 h before the termination of the culture. Cells were then harvested on glass fiber filters using a cell harvester (Filtermate 196; Packard Bioscience Co., CT) and radioactivity was measured with MicroScint™ O solution (PerkinElmer, MA) using a Top Count® NXT™ (Packard Bioscience) or Wallac 1450 MicroBeta® TriLux (PerkinElmer) microplate scintillation counter.

### CFSE dilution assays

In some experiments, responder cells were stained with CFSE (Sigma Aldrich) prior to *in vitro* culture. Briefly, cells were resuspended in OPTI-MEM (Invitrogen) at a concentration of 2×10^6^ mL^−1^ and incubated with 500 nM CFSE for 10 min at 37°C before adding MLR medium. The cells were then washed in MLR medium twice, incubated for 5 min at 37°C, washed again and resuspended in MLR medium. At the termination of MLR and Con A cultures, cells were harvested and washed in phosphate buffered saline before labeling with monoclonal antibodies (mAb) and flow cytometric analysis.

### Intracellular staining and flow cytometry

The following mouse anti-rat mAb (conjugated in our own lab unless stated otherwise; W3/25 and OX antibodies were a kind gift from A. Neil Barclay, Oxford, UK) were used for flow cytometric analysis: FITC-conjugated W3/25 (anti-CD4), PE-conjugated G4.18 (anti-CD3; BD Biosciences), Alexa Fluor® 647-conjugated OX-38 (anti-CD4), biotin-conjugated OX-8 (anti-CD8) or OX-39 (anti-CD25) followed by secondary staining with PerCP-conjugated Streptavidin (BD Biosciences). In some experiments, 50 µM propidium iodide (Sigma Aldrich) was added to stained cells before analysis.

For intracellular FoxP3 staining, stimulated LNC were harvested after 3 d of culture, stained with mAb, fixed with fixation/permeabilization buffers (eBioscience, CA) and stained with APC-conjugated rat anti-mouse/rat FJK-16s mAb (anti-FoxP3; eBioscience) according to the manufacturer's guidelines. For intracellular IFNγ staining, LNC were stimulated with Con A (5 µg mL^−1^; Sigma Aldrich) for 24 h, and Brefeldin A (10 µg mL^−1^; Sigma Aldrich) was added 4 h before termination of the culture. Triplicate wells were pooled and cells stained with FITC-conjugated G4.18 (anti-CD3; BD Biosciences), fixed with 4% paraformaldehyde, permeabilized with 0.5% saponin, and stained with PE-conjugated mouse anti-rat IFNγ mAb (BD Biosciences).

For phenotypic characterization of MSC, cells were labeled with the following conjugated mouse anti-rat mAb: PE-conjugated TLD-3A12 (anti-CD31) or OX-7 (anti-CD90); FITC-conjugated TH9 (anti-CD59); PE-Cy5 conjugated OX-1 (anti-CD45; all from BD Biosciences). For two-step immunostaining, cells were first incubated with supernatant of OX-26 (anti-CD71), or purified OX-18 (anti-RT1-A, pan-MHC class I) or OX-6 (anti-RT1-B/D, pan-MHC class II; all our own), followed by secondary PE-conjugated donkey anti-mouse IgG (Jackson ImmunoResearch, UK); or with TP-503 (polyclonal rabbit anti-rat CXCR4; Torrey Pines Biolabs, NJ) followed by secondary FITC-conjugated anti-rabbit IgG (BD Biosciences). PE-conjugated mouse IgG_1_ (BD Biosciences) and FITC-conjugated mouse IgG_2a_ (DAKO, Denmark) were used as isotype controls. Cells were analyzed on a FACSCalibur™ or FACSCanto™ flow cytometer (BD Biosciences) using CellQuest™ software (BD Biosciences). FACS data were further analyzed using FlowJo™ software (TreeStar Inc., OR).

### Cytokine assays

For cytokine measurements, 10×10^6^ responder and 10×10^6^ stimulator cells were co-cultured for 6–7 d in MLR medium together with specified numbers of MSC in upright 25 cm^2^ culture flasks (Nunc). Cytokine levels were analyzed using the Bio-Plex™ Rat Cytokine 9-Plex A Panel (Bio-Rad, CA) according to the manufacturer's protocol. Concentrations were determined on the basis of a standard curve using defined reference samples which were assayed (Luminex xMAP® Technology; Bio-Rad) in parallel.

### Experimental BMT and GvHD monitoring

Allogeneic stem cell transplantations were adapted from a previously described protocol [49]. BM cells were aspirated from femurs and tibias of 8-12 w-old male and female PVG.7B rats, filtered through cell strainers (BD Biosciences) and purified by density gradient centrifugation (Nycoprep™ 1.077A; Medinor). T cells were removed using magnetic pan-mouse IgG-coated Dynabeads® (Invitrogen Dynal, Norway) coated with OX-19 (anti-CD5) and R73 (anti-αβ T cell receptor) mAb. The number of CD3^+^ cells was thus reduced at least fivefold to less than 0.25% of total BM cells (data not shown). For DLI, LNC (of which approximately 60% were CD3^+^ T cells, data not shown) were obtained from mesenteric and cervical lymph nodes of 10-12 w-old male and female PVG.7B rats by filtering through cell strainers (BD Biosciences). The CD3^+^ T cell contents of the BM graft and DLI were controlled by flow cytometric analysis (not shown) after staining with PE-conjugated G4.18 (anti-CD3; BD Biosciences) mAb.

Male BN rats were transplanted at 9 w of age with 30×10^6^ T cell-depleted PVG.7B BM cells by intravenous injection shortly after receiving 9.0 Gy total body irradiation (Gammacell® 3000; MDS Nordion) under anesthesia. 14 days post transplantation, GvHD was invoked by DLI of the specified doses of PVG.7B LNC. MSC were harvested, washed and resuspended in phosphate buffered saline at a cell density of 1–2×10^6^ per mL for injection at the specified time points. Transplanted animals were frequently and carefully monitored, and were scored weekly for disease symptoms using a protocol by Cooke *et alia* [33]. In this model, cachexia, severe kyphosis, ruffled fur, skin flaking and lesions and alopecia were predictive of GvHD. Rats suffering from lethal GvHD were euthanized with CO_2_ at defined humane end points (GvHD score exceeding 8; body weight below 150 g). Donor BM engraftment was evaluated both by clinical observation (weight gain, anemia) and flow cytometry using HIS41 (anti-rat CD45) mAb (a kind gift from Jaap Kampinga, Groningen, The Netherlands) specific for the RT7.2 allele to identify PVG.7B donor leukocytes. 7 rats (8.9%) of a total of 79 recipients failed to engraft and were excluded from the analysis.

### Statistical analysis

Normal distribution of data was assumed and tested using Shapiro-Wilk's test. Student's *t* test (paired, two-tailed) was used to evaluate the probability of differences between samples. For *in vivo* experiments, overall survival was compared by the log-rank test and GvHD scores by Student's *t* test (unpaired, two-tailed) for group differences. Analysis was performed using SPSS® software version 17.0 (SPSS Inc., IL) with a *P* value below 0.05 deemed as significant.

## Supporting Information

Figure S1 **Mycoplasma-infected MSC reduce the numbers of T regulatory and live cells in mitogen-stimulated lymphocyte cultures.** Mycoplasma-infected MSC (PVG.7B; gray bars) were added at the start of a Con A stimulated culture of PVG.7B LNC at the indicated dilutions (log [MSC:LNC]). The relative frequency of (**A**) CD4^+^CD25*^hi^*FoxP3^+^ T regulatory (T_reg_) cells and (**B**) live cells (propidium iodide*^low^*) were determined by flow cytometry after 3 d of co-culture. Cell death was increased when mycoplasma-infected MSC were present. In panel A, the mean values plus the standard error of the mean of triplicates as well as the test statistics for cell frequencies compared to the positive control are shown. Data are representative of three independent experiments.
(TIF)

Figure S2 **Morphology, differentiation potential and phenotype of mycoplasma-infected rat BM-derived MSC.** Light microscopy of cell culture, differentiation assays and flow cytometric staining of MSC infected with *M. hyorhinis*. (**A**) MSC from PVG BM appear as fibroblast-like spindle-shaped cells that adhere to plastic *in vitro* (third passage). MSC have the capacity to differentiate into adipocytes (**B**) as shown by staining of neutral lipids in fat vacuoles with Oil Red O and osteocytes (**C**) by staining areas of calcification with Alizarin Red. (**D**) Surface expression of CD31 (PECAM-1), CD45 (CLA), CD59 (MAC inhibitor), CD71 (transferrin receptor), CD90 (Thy-1) and CXCR4 as well as MHC-I (RT1-A) and MHC-II (RT1-B/D) on MSC. Histograms show the relative intensity of surface antigen (solid lines) compared to isotype controls (filled) by flow cytometric staining. Mycoplasma-infected PVG.1U MSC form adipocytes (**E**) and osteocytes (**F**) under culture conditions inducing differentiation (cf. Materials and Methods) and remain multipotent (**G**, **H**) after clearing the infection with Mynox reagent. (**A**) Original magnification 100×, (**B**, **C**) 40×, (**E–H**) 200×.
(TIF)

Figure S3 **MSC inhibition of MLR is not reversed by addition of exogenous IFNγ nor by anti-IL-6 antibody.** (**A**) A panel of cytokines was measured in the medium supernatant of a confluent culture of infected MSC (PVG) 20–24 h after medium was replaced. MSC constitutively secrete IL-6, while other cytokines were not detectable. (**B**) Inhibition of proliferation in allogeneic MLR at 1∶1000 MSC:LNC ratio could not be reverted by addition of anti-rat IL-6 mAb (αIL-6; 2 µg mL^−1^) or recombinant rat IFNγ (500 U mL^−1^) at the start of co-culture. Representative data from two independent experiments are shown as mean values plus one standard error of the mean of (**A**) triplicate and (**B**) quadruplicates tests.
(TIF)

Figure S4 **Rats suffering from GvHD are not rescued by repeated injections of mycoplasma-infected MSC.** MSC from two different cell lines subsequently found to be infected with *M. hyorhinis* were injected in rats suffering from experimental acute GvHD. Irradiated BN recipients were transplanted with 30×10^6^ T cell-depleted donor PVG.7B BM cells (▵) and received a DLI 14 d later (▴) of graded doses of either (**A**, **B**) 2.5×10^6^ or (**C**, **D**) 1.5×10^6^ donor PVG.7B LNC. Two rats that received BM cells only (no DLI) and did not develop GvHD are also shown in panel C. 0.5–1×10^6^ MSC from PVG.1U (**A**, **B**) or 1–2×10^6^ MSC from PVG (**C**, **D**) were injected either repeatedly on 0, 7, and 14 d (bold line, 3× MSC) or once on 14 d (solid line, 1× MSC) after DLI. Control rats received no MSC (dashed line, no MSC). (**A**, **C**) Cumulative survival is depicted as Kaplan-Meier plots. (**B**, **D**) GvHD symptoms, including relative change in body weight, were assigned discrete values using a semi-quantitative scoring table adapted from Cooke *et alia* [33]. GvHD scores are shown together with the median (horizontal line) at 7 d and 14 d after DLI, respectively. The respective MSC treatment protocols had no statistically significant effect on overall survival nor the GvHD score of rats treated with 3× MSC (•) or 1× MSC (•) compared to the controls (°). Data were pooled from (**A**, **B**) two and (**C**, **D**) four individual experiments.
(TIF)
