# Supplementary figures and images for: Mycoplasma Contamination Revisited: Mesenchymal Stromal Cells Harboring Mycoplasma hyorhinis Potently Inhibit Lymphocyte Proliferation In Vitro
Source: PLoS One. 2011 Jan 11;6(1):e16005. doi: 10.1371/journal.pone.0016005 (PMC3019172; doi:10.1371/journal.pone.0016005)

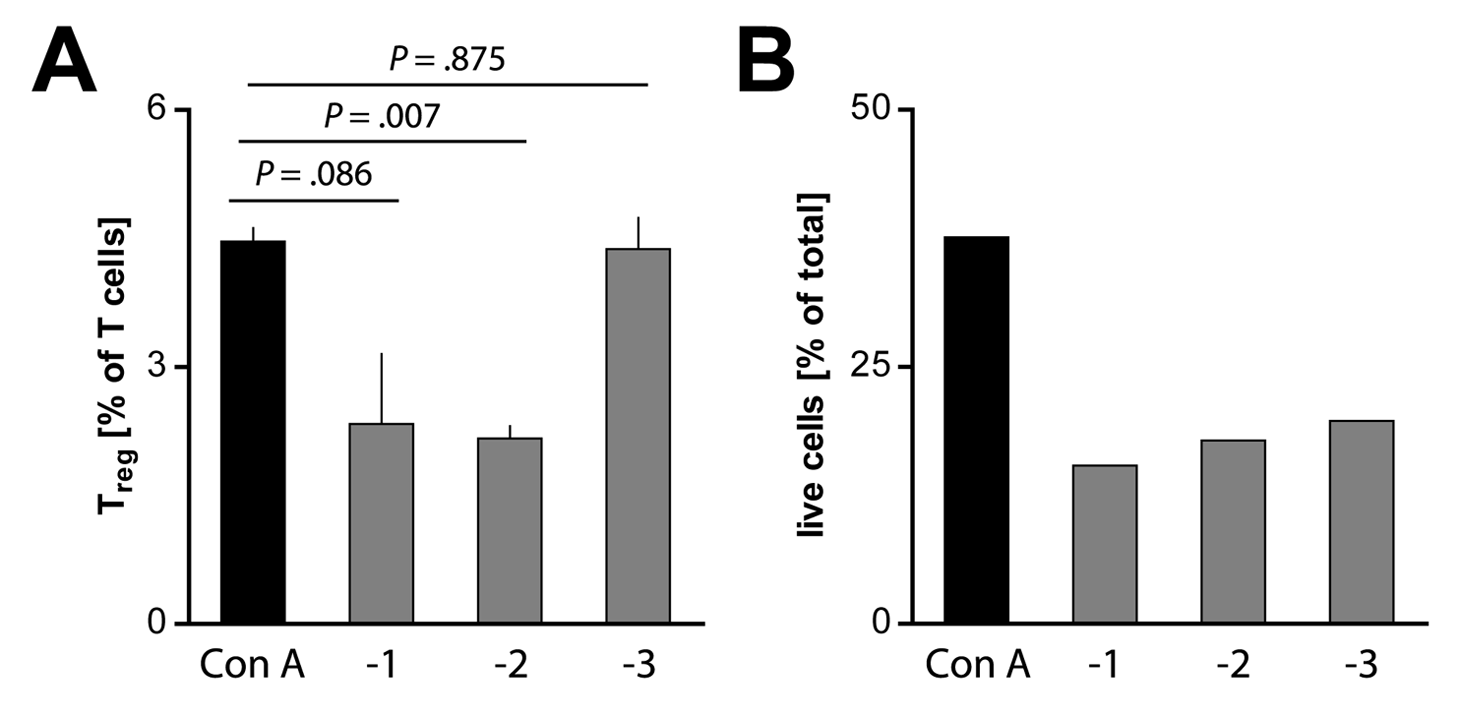

Supplement: Figure S1 — Mycoplasma-infected MSC reduce the numbers of T regulatory and live cells in mitogen-stimulated lymphocyte cultures. Mycoplasma-infected MSC (PVG.7B; gray bars) were added at the start of a Con A stimulated culture of PVG.7B LNC at the indicated dilutions (log [MSC:LNC]). The relative frequency of (A) CD4+CD25hiFoxP3+ T regulatory (Treg) cells and (B) live cells (propidium iodidelow) were determined by flow cytometry after 3 d of co-culture. Cell death was increased when mycoplasma-infected MSC were present. In panel A, the mean values plus the standard error of the mean of triplicates as well as the test statistics for cell frequencies compared to the positive control are shown. Data are representative of three independent experiments. (TIF) [file pone.0016005.s001.tif]

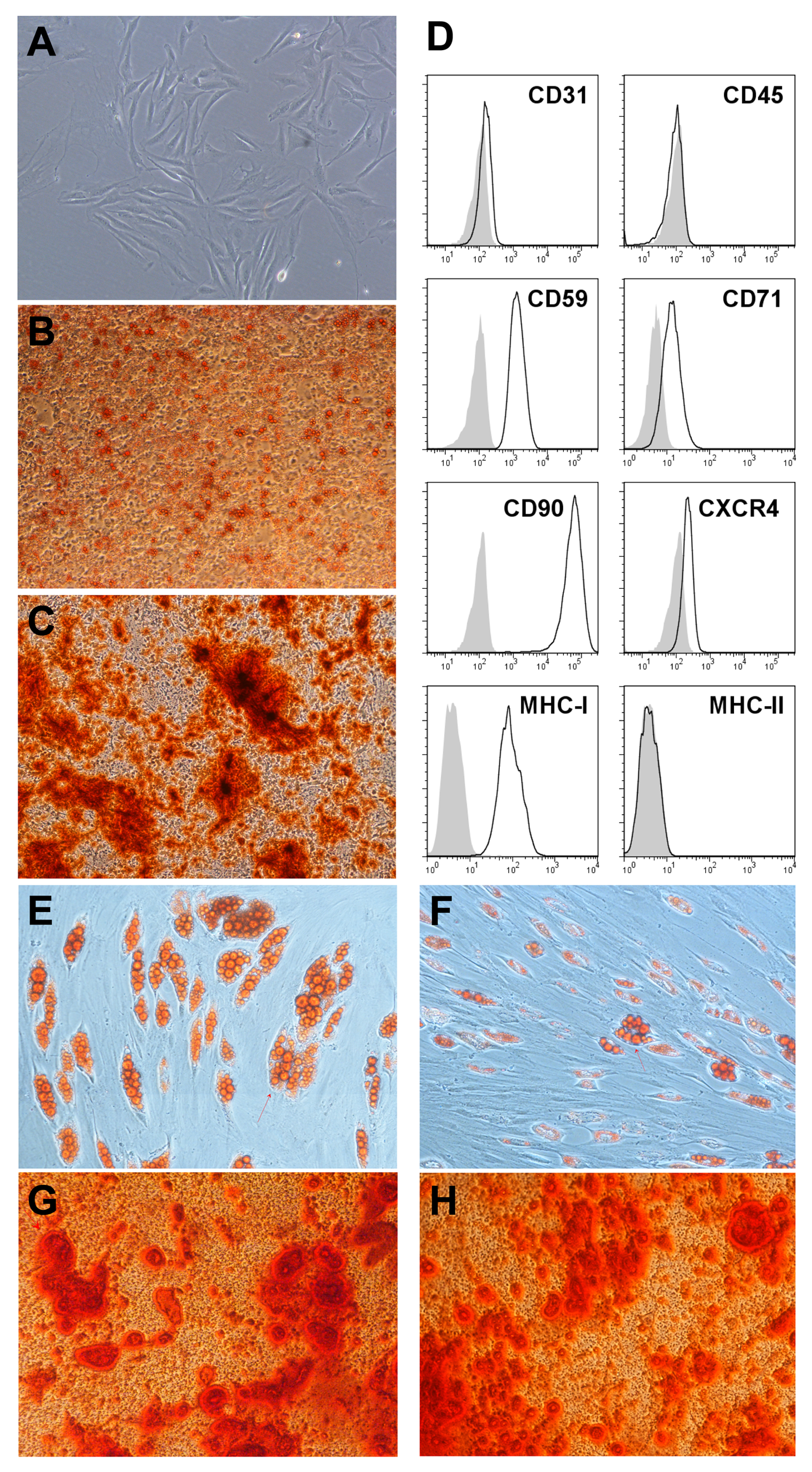

Supplement: Figure S2 — Morphology, differentiation potential and phenotype of mycoplasma-infected rat BM-derived MSC. Light microscopy of cell culture, differentiation assays and flow cytometric staining of MSC infected with M. hyorhinis. (A) MSC from PVG BM appear as fibroblast-like spindle-shaped cells that adhere to plastic in vitro (third passage). MSC have the capacity to differentiate into adipocytes (B) as shown by staining of neutral lipids in fat vacuoles with Oil Red O and osteocytes (C) by staining areas of calcification with Alizarin Red. (D) Surface expression of CD31 (PECAM-1), CD45 (CLA), CD59 (MAC inhibitor), CD71 (transferrin receptor), CD90 (Thy-1) and CXCR4 as well as MHC-I (RT1-A) and MHC-II (RT1-B/D) on MSC. Histograms show the relative intensity of surface antigen (solid lines) compared to isotype controls (filled) by flow cytometric staining. Mycoplasma-infected PVG.1U MSC form adipocytes (E) and osteocytes (F) under culture conditions inducing differentiation (cf. Materials and Methods) and remain multipotent (G, H) after clearing the infection with Mynox reagent. (A) Original magnification 100×, (B, C) 40×, (E–H) 200×. (TIF) [file pone.0016005.s002.tif]

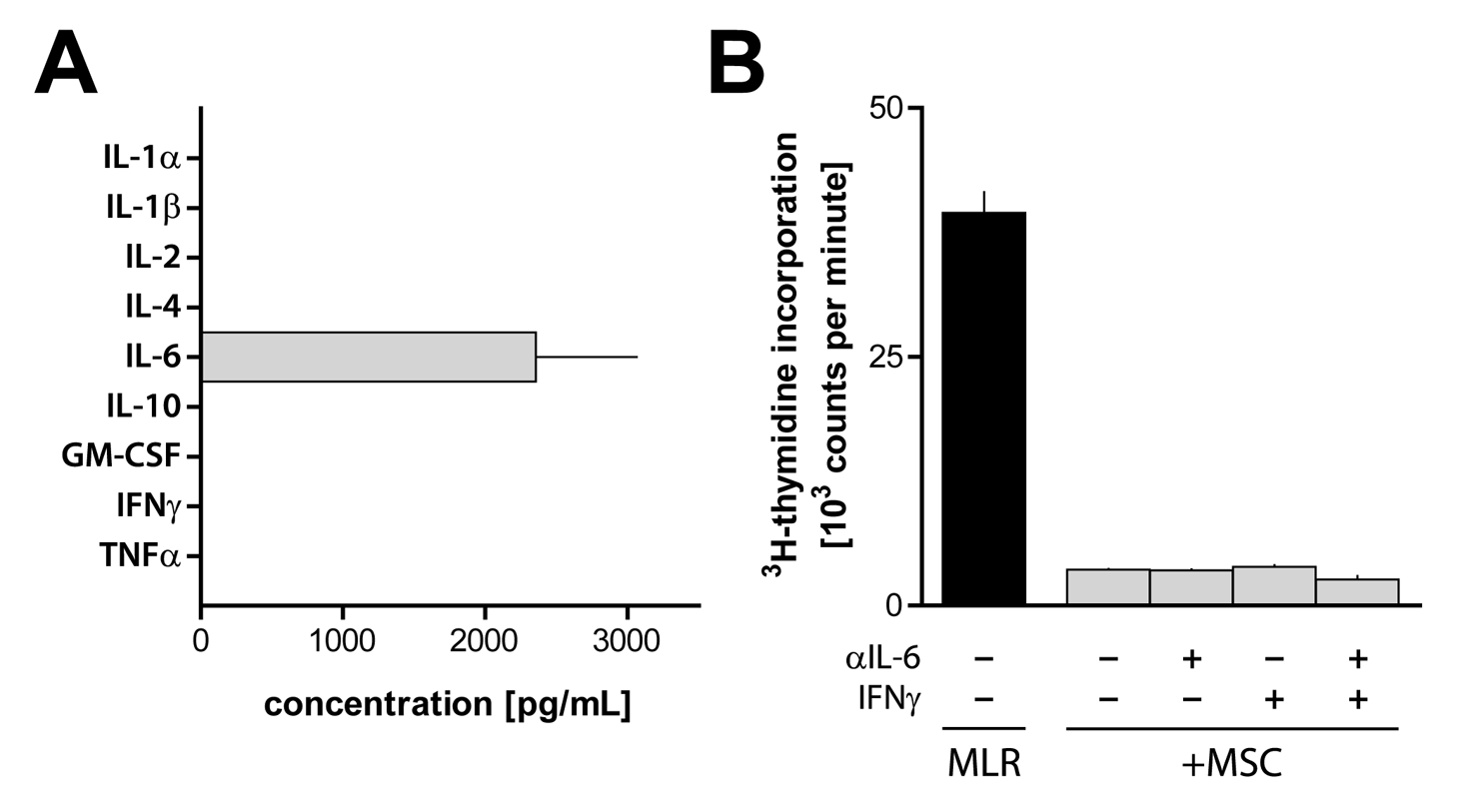

Supplement: Figure S3 — MSC inhibition of MLR is not reversed by addition of exogenous IFNγ nor by anti-IL-6 antibody. (A) A panel of cytokines was measured in the medium supernatant of a confluent culture of infected MSC (PVG) 20–24 h after medium was replaced. MSC constitutively secrete IL-6, while other cytokines were not detectable. (B) Inhibition of proliferation in allogeneic MLR at 1∶1000 MSC:LNC ratio could not be reverted by addition of anti-rat IL-6 mAb (αIL-6; 2 µg mL−1) or recombinant rat IFNγ (500 U mL−1) at the start of co-culture. Representative data from two independent experiments are shown as mean values plus one standard error of the mean of (A) triplicate and (B) quadruplicates tests. (TIF) [file pone.0016005.s003.tif]

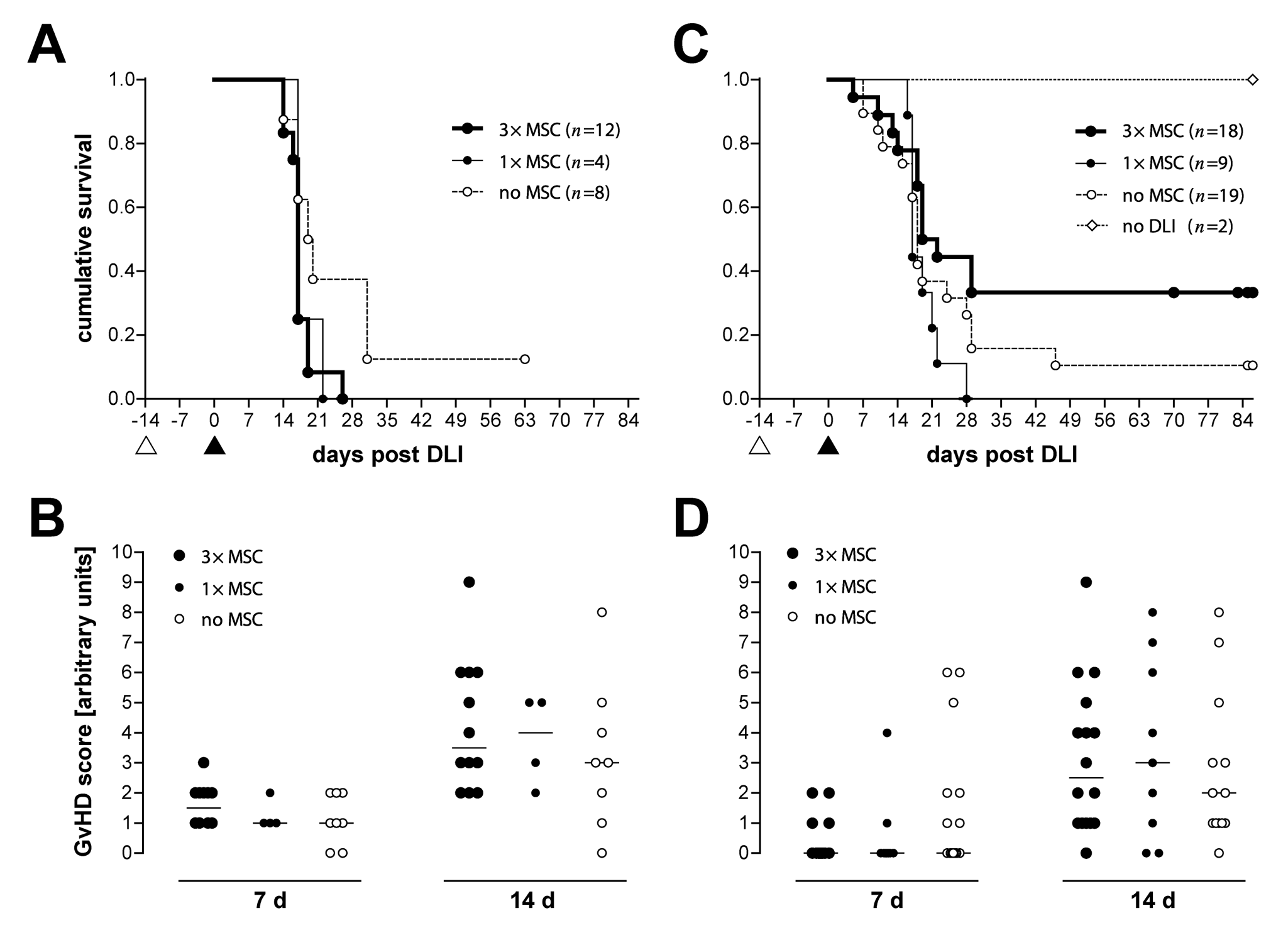

Supplement: Figure S4 — Rats suffering from GvHD are not rescued by repeated injections of mycoplasma-infected MSC. MSC from two different cell lines subsequently found to be infected with M. hyorhinis were injected in rats suffering from experimental acute GvHD. Irradiated BN recipients were transplanted with 30×106 T cell-depleted donor PVG.7B BM cells (▵) and received a DLI 14 d later (▴) of graded doses of either (A, B) 2.5×10^6^ or (C, D) 1.5×10^6^ donor PVG.7B LNC. Two rats that received BM cells only (no DLI) and did not develop GvHD are also shown in panel C. 0.5–1×10^6^ MSC from PVG.1U (A, B) or 1–2×10^6^ MSC from PVG (C, D) were injected either repeatedly on 0, 7, and 14 d (bold line, 3× MSC) or once on 14 d (solid line, 1× MSC) after DLI. Control rats received no MSC (dashed line, no MSC). (A, C) Cumulative survival is depicted as Kaplan-Meier plots. (B, D) GvHD symptoms, including relative change in body weight, were assigned discrete values using a semi-quantitative scoring table adapted from Cooke et alia [33]. GvHD scores are shown together with the median (horizontal line) at 7 d and 14 d after DLI, respectively. The respective MSC treatment protocols had no statistically significant effect on overall survival nor the GvHD score of rats treated with 3× MSC (•) or 1× MSC (•) compared to the controls (°). Data were pooled from (A, B) two and (C, D) four individual experiments. (TIF) [file pone.0016005.s004.tif]
